# Fischer’s *Lexicon of Slavic beliefs and customs*: a previously unknown contribution to the ethnobotany of Ukraine and Poland

**DOI:** 10.1186/s13002-015-0073-8

**Published:** 2015-12-24

**Authors:** Monika Kujawska, Łukasz Łuczaj, Joanna Typek

**Affiliations:** Institute of Ethnology and Cultural Anthropology, University of Łódź, ul. Pomorska 149/153, 90-236 Łódź, Poland; Department of Botany, Institute of Applied Biotechnology and Basic Sciences, University of Rzeszów, Werynia 502, 36-100 Kolbuszowa, Poland; Institute of History, Faculty of Sociology and History, University of Rzeszów, ul. Rejtana 16 C, 35-959 Rzeszów, Poland

**Keywords:** Historical ethnobotany, Archival data, Medicinal plants, Ritual plants, Western Ukraine

## Abstract

**Background:**

Historical ethnobotanical studies are important, even if they are only descriptive, because they help to throw light on the missing chains needed for diachronic analysis. However, the documentation of traditional uses of plants in some countries, e.g. Ukraine, is still fragmentary. The aim of this contribution is to fill the gap and present a portion of the data set, from western Ukraine, which was collected by Adam Fischer, a Polish ethnographer from Lviv, in the 1930s. These data were originally gathered to be published in the first part of the *Lexicon of Slavic beliefs and customs,* dedicated to plant uses in traditional Slavonic culture. The idea of writing the *Lexicon* arose in 1929 during the I Congress of Slavic Philologists in Prague and was intended to be a joint international enterprise, but has never actually been fulfilled.

**Methods:**

In this article we used information from south-eastern Poland at that time – nowadays western Ukraine, collected in four provinces, 11 counties and 28 localities by Fischer’s collaborators. The majority of the information was accompanied by voucher specimens, which were determined by botanists at the Jan Kazimierz University. These data are still unpublished and stored on filecards in the archives of the Polish Ethnological Society in Wrocław, Poland. In our analysis we applied two indices: one to measure general plant versatility – Use Value, and another regarding medicinal plants – Relative Importance Value.

**Results:**

In total, 179 plant taxa used in peasant culture in the western Ukraine in the 1930s were registered. The species which achieved the highest Use Values were: *Achillea millefolium*, *Allium sativum*, *Vinca minor*, *Hypericum* sp. and *Juniperus communis*. Among the collected plant names, Polish names dominate (59 %) over clearly Ukrainian and Ruthenian ones (31 %). The remaining 10 % of names were of unclear origin or could have been used by both groups. The most salient use categories were medicinal, followed by ritual – chiefly plants used in church ceremonies, followed by animal wellbeing (veterinary and fodder). However we learn very little about plant management in the peasant culture from the data set.

**Conclusions:**

Analysis of the archival data threw new light on plant use and management in the Galicja region in the interwar period. It also increased our understanding of the central role of plants in spheres such as folk medicine, church ceremonies and animal wellbeing.

## Background

### Ethnobotanical studies concerning Ukraine

Although ethnobotany is a science which has already amassed a huge amount of field studies and is now largely preoccupied with the theories, principles and processes which explain the field information and observations, the documentation of traditional uses of plants in some countries is still very fragmentary. Even in Europe, the level of saturation with ethnobotanical studies is very uneven. In some countries either archival sources or contemporary field data are very abundant, e.g. [1–3]. At the other extreme are countries, such as Ukraine, where such studies are relatively scarce. In this paper we present archival data concerning western Ukraine, gathered by Adam Fischer (1889-1943), a Polish ethnographer from the Jan Kazimierz University in Lviv (Polish: Lwów, German: Lemberg, Russian: Lvov).

The ethnobotany of Ukraine, one of the largest and most populous European countries, is a neglected field. It is actually difficult to define this issue due to the changing borders in this part of Europe and the lack of a Ukrainian state until a few decades ago. The territory of present day western Ukraine was:

1. an easternmost zone of interest for Polish 19th/20th century ethnographers, due to the fact that it used to be part of the Polish-Lithuanian commonwealth until the late 18th century, and after 1918 became once more a part of the Republic of Poland. Ukrainian peasants were thus seen as a natural object of study for a Polish ethnographer, though in many areas Polish inhabitants constituted a large part of the population of western Ukraine. Actually, Ukrainian peasant culture was often idealized as more “unpolluted” and “authentic”, especially during the 19th century Romanticism period [4].
2. an active arena for local Ukrainian patriotic ethnographers. Their efforts, however, concerned mainly the protection of language and songs. This results in a rich folklore literature and a lack of serious works of an ethnobotanical character. To our knowledge, Russian ethnographic literature does not contain any ethnobotanical studies concerning the central and eastern part of Ukraine which, for a few centuries, belonged to the Russian Empire.

All in all, most older ethnobotanical data come from research published in Polish [5–12]. One of the most important Polish-language contributions to the ethnobotany of Ukrainian people is the work of Talko-Hryncewicz [11], a physician and physical anthropologist who recorded folk medicine including plant medicines from a few places in central and western Ukraine.

Many unpublished materials concerning Ukraine are also to be found in responses to Rostafiński’s questionnaire of 1883 [13, 14]. Only uses and names of wild food plants and mushrooms have been published so far [15–18]. Data on wild edible plants in three counties of the Hutsul and Pokucie areas in the Ukrainian Carpathians were also gathered by Adam Fischer in 1934, in an ethnographic questionnaire sent to a few hundred school teachers [19]. This subject was analyzed in a separate publication [20].

Paradoxically, taking into account the scarcity of ethnobotanical studies in Ukraine, the issue of local plant names is a well studied topic, as is the case in Russia. Contemporary knowledge was synthesized by Kobiv [21] in his *Dictionary of Ukrainian plant names*. Ukrainian plant names related to Ukrainian ethnonyms are also analyzed in the works by the Russian ethnolinguist Valeriia Kolosova [22].

Over the last few years, growing interest in the ethnobotany of Ukraine may be observed – a few papers have appeared on this topic: two concerning the territory of western Ukraine [23, 24] and another two the Maramureş region in Romania, adjacent to Ukraine and inhabited by a Ukrainian minority [25, 26]. A few years ago a monograph of plants involved in the folk beliefs of Ruthenian-Ukrainians in Slovakia was also published [27].

### The contribution of Adam Fischer: plants in folk beliefs and customs

Adam Robert Fischer (1889–1943) was a Polish philologist, folklorist and ethnographer. From 1924 he was a professor at the Department of Ethnography, and from 1934/1935 the Dean of the Faculty of Humanities at the University of Jan Kazimierz in Lwów (now Lviv) [28]. Fischer dedicated most of his life to the development of the Polish Ethnological Society, as underlined in the words of Czekanowski: “Ethnological Society – it was Fischer and only Fischer” [28]. He spent 33 years working as the editor-in-chief of *Lud* – the oldest Polish ethnological journal, which he kept to a modern European level [29]. The legacy of Professor Adam Fischer contains a rich collection of articles, books and unpublished materials. These materials are stored in archives and consist of manuscripts, surveys, lectures and scientific correspondence [30]. The collection, which is now owned by the Polish Ethnological Society, was transported from Lviv after World War II by the professor’s family [31].

Fischer’s largest unpublished ethnobotanical work was the result of taking part in an international project called *Lexicon of Slavic beliefs and customs.* The idea for this work arose during the I Congress of Slavic Philologists in Prague in 1929. Its first part was to be focused on plants in folk culture. In order to accomplish this task, five editors were appointed from five Slavic countries: Christo Vakarelski from Sofia, Veselin Cajkanovic from Belgrade, Karel Chotek from Prague, Adam Fischer from Lviv and Dmitri Konstantinovich Zielenin from Leningrad. The initiator and main editor was Edmund Schneeweis from Prague [32, 33]. The *Lexicon* was to be published by the Walter de Gruyter editorial house. The material for this enterprise was supposed to be collected during fieldwork and compiled within a year. We do not know how the work on *Lexicon* was developed in other countries, but Adam Fischer launched his field campaign just after the Congress. The same year, in *Lud,* he published a call to all ethnographers and persons interested in collecting information from peasants in the whole of the Polish territory on plant beliefs and uses [34]. He asked for unpublished notes, as well as contributions published in the local press. In the same article, he elucidated topics which should be taken into account while conducting fieldwork:

1. Local plant names and possible etymologies
2. Practical application and use of plants in everyday life, such as: food, construction material, cloths, dyeing agents, medicines and poisons
3. Plants with special magical powers, plants in love lore, bestowed with extraordinary virtues enabling the user to ascend into the air or to become invisible
4. Plants with symbolic significance in rituals and ceremonies, such as weddings, funerals and “chodzenie z maikiem”.
5. Plants as decorative motifs present in houses, on cutlery, clothing, embroidery, cutouts and Easter eggs
6. Toys made from plants, e.g. cockerels, pipes, ropes; caps made of rushes, poppers made of elder, necklaces from rowan, fans, straws etc.
7. Plants in stories and folk songs

In addition to this questionnaire, he enclosed an alphabetical list of 260 plant species according to Polish common names, with Latin names in brackets. The list also contained generic names (e.g. berries) and covert categories (e.g. trees, vegetables). Fischer explained that the list was intended as a prompt, facilitating fieldwork, and it was indeed not complete but enclosed only the “most significant taxa” [34]. He added that collectors should make notes of other species too, if they cropped up during the interviews. He stressed that the information should always include the place where the research was conducted and the name of the informant. Finally, he asked that all the gathered material be sent to his office at the Jan Kazimierz University in Lviv.

That call did not produce much effect, however, and it was repeated in 1930 in a tourism oriented magazine for young people, *Orli Lot*, in which Polish ethnographers announced their research and asked for the help of the Koła Krajoznawcze [Tourist Clubs], especially if their research was based on field questionnaires. The article contained the same information as the one in *Lud*, but the author did not enclose the plant list this time [35]. Fischer repeated his appeal one more time in *Orli lot* in 1934. In a short article, entitled *Reminder about a plant questionnaire*, he wrote that the response to his call was rather poor, but he acknowledged the contribution of a few Tourist Clubs from Żywiec (two different ones), Zamość and Bochnia – all from southern Poland, and one from Czarnków in the north-western part of the country. Interestingly, Fischer repeated his request to continue the collection of data but this time he gave different instructions for gathering plant information. He asked for plants used in: 1) folk medicine, 2) magic, 3) as dyeing agents, 4) as children’s toys, 5) as wild gathered food, 6) stories related to plants, 7) folk plant names [36]. We can only presume that this change in the formulation of questions was influenced by the already received material, and perhaps these domains yielded the most salient or interesting results. However, we have no access to any correspondence by Fischer or other researchers to confirm this assumption. In this article, Fischer paid attention for the first time to the importance of gathering corresponding plant material – voucher specimens, which after being determined by botanists would be returned to the collectors. He gave practical instructions on how to combine voucher specimens with plant names and related information, in order not to mix up the material.

It can be easily figured that information gathering for the first part of the *Lexicon* was extended till the mid-1930s. Altogether, Fischer received information and voucher specimens from eight Tourist Clubs and 20 different field collaborators – some of them working independently, such as the ethnographer Sebasitan Flizak from Sanok (in the Lviv province at that time). From his letters to Fischer we can estimate the number of vouchers he sent to the Jan Kazimierz University (Archives of the Polish Ethnological Society, sign. 543). Fischer thought, however, that the original field material was not sufficient to prepare the *Lexicon* entries, therefore he complemented the information on plant taxa with published sources, starting from Rennaissance herbals and finishing with contemporary ethnographic articles and books. The whole list of references used during the work on the *Lexicon* is stored in the Archives of the Polish Ethnological Society in Wrocław (sign. 356).

What happened to the material destined for publication in the first part of the *Lexicon*? In a letter directed to Dmitri Konstantinovich Zielenin from 20th of December 1940, Fischer asks *inter alia* about progress on the *Lexicon*: “We are both co-editing a volume led by Professor E. Schneeweis, entitled *Handwörterbuch des slawischen Volsglaubens und Volksbrauchs*. You, my dear colleague, were to prepare the part dedicated to eastern Slavs, and myself about the Polish territory. Have you sent the whole manuscript to Prague yet? Is this thing being printed? I have sent the manuscript with entries up to the letter K (medicinal beliefs related to plants, etc.) but then the war broke out and I lost contact with the editors, so I do not know whether this editorial board exists or has been suspended *ad pacem*” [29]. In fact, Fischer’s unpublished manuscript, written in German, has been stored in the Archives of the Masaryk Institute in Prague until now. The fate of other parts of the *Lexicon* remains unknown. In Zelenin’s references there is no information about any such publication [29].

The aim of this contribution is to describe and analyse a portion of the data set collected for the editing of the *Lexicon*. As we found the whole material to be vast, we divided it into parts for further analysis, according to the regional key corresponding to the country the materials belong to now (e.g. Poland, western Ukraine, western Belarus and Lithuania). Here we concentrate on the material from western Ukraine, which was collected among both Polish and Ukrainian peasants. The Results section is a faithful translation of the field information, with minimal *etic* inference, such as use categories introduced by us, and two basic indices to measure culturally important species. In the Discussion section, however, we aimed to look at “old things with new eyes” and interpret the data according to our understanding of ethnobotanical processes in the study domains and in this particular region.

## Methods

### Study area

**Fig. 1:**
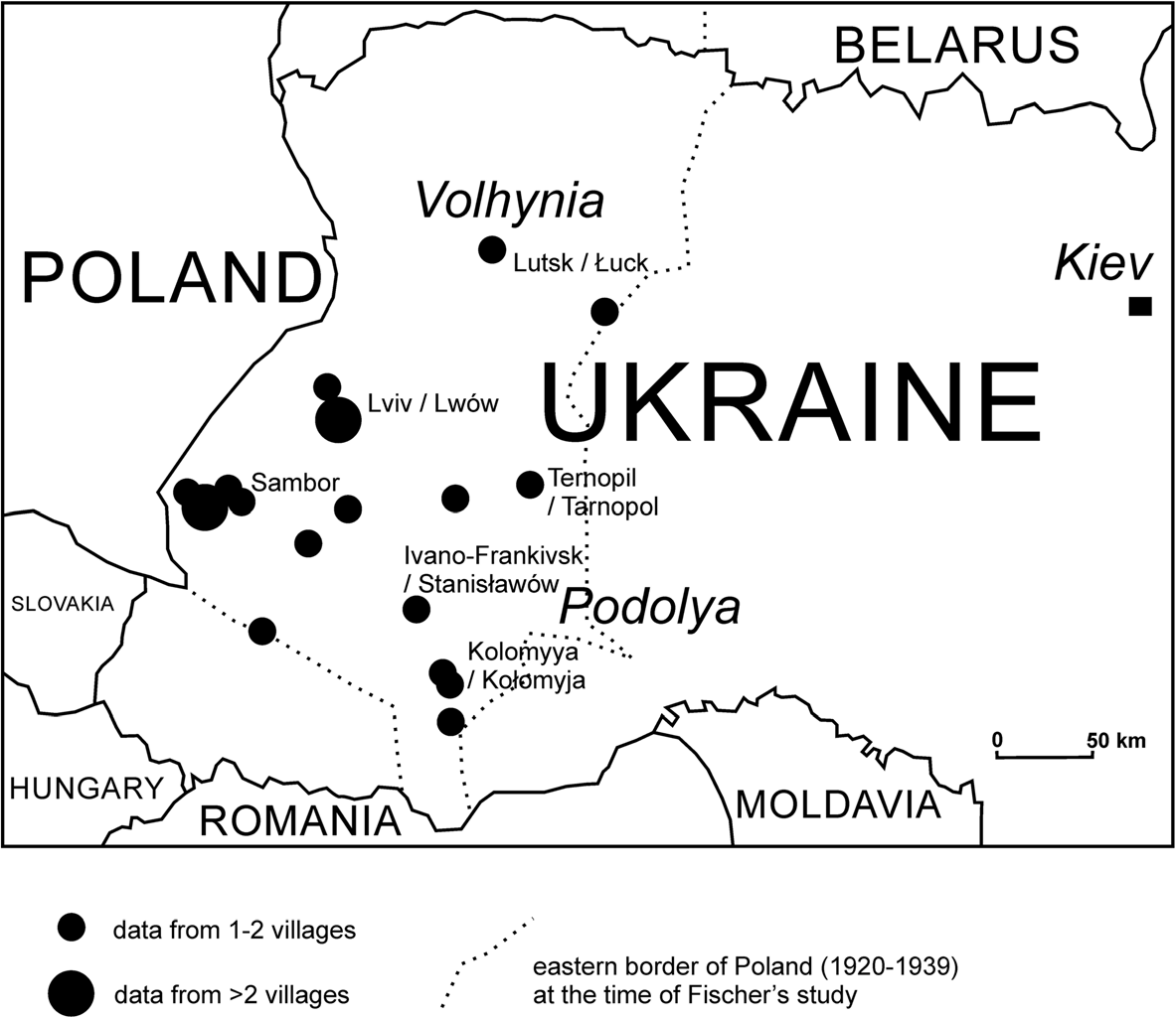
Distribution of the study localities in the 1930s in Eastern Galicja (Poland), now western Ukraine

Fischer was interested in the whole area of pre-World War II Poland, which also includes present areas of western Belarus, western Ukraine and parts of Lithuania. The data presented here come from most regions of western Ukraine. In the interwar period they embraced four provinces: Lwowskie (now Львівська область), Stanisławowskie (now Івано-Франківська область), Tarnopolskie (Тернопільська область) and Wołyńskie (now Волинська область). A few pieces of information came from the Podole (Podolia) region (a larger historical region in Ukraine). Both Polish and Ukrainian names are reported by Fischer’s informants. In many cases it is possible to distinguish the ethnicity of names, and sometimes not: it is possible that some of the names were used by both ethnic groups (Fig. 1).

The present area of western Ukraine was a multicultural, multi-ethnic area in Fischer’s times. On the whole, Ukrainians or Ruthenians predominated (the latter name was used to describe people speaking dialects which can be classified as Ukrainian but without a Ukrainian ethnic identity, which was often the case in rural areas). They were the original inhabitants, living mainly in the countryside. Poles were the second largest ethnic group, and locally, especially around Lwów, Sambor, Stanisławów and Tarnopol, they even constituted over half of the population. Their co-domination was the result of a few hundred years of migration from ethnically Polish areas of the Polish-Lithuanian Commonwealth, coupled with recent migration in the years between the World Wars, encouraged by the Polish government. Ashkenazi Jews were another important ethnic group, settled mainly in towns [37, 38].

After World War II, due to the shift of borders in Poland imposed by Stalin and accepted by the Treaty of Jalta (1945), around two million Poles were re-settled from the eastern part of the country, mainly to western and northern Poland, to the areas taken by Poland from Germany. Additionally, a few hundred thousand Poles were murdered during ethnic cleansings performed by Ukrainians, particularly in the Volhynia area. This made western Ukraine an ethnically Ukrainian region. Later, however, during the Soviet times, many Russians or Russian-speaking Ukrainians settled in western Ukraine as well. Thus the ethnobotany we are dealing with in this article concerned a country which was very different ethnically (mixed Ukrainian and Polish, with Jewish admixtures), than the present day western Ukraine (Ukrainian with Russian admixtures).

### Data gathering

**Fig. 2:**
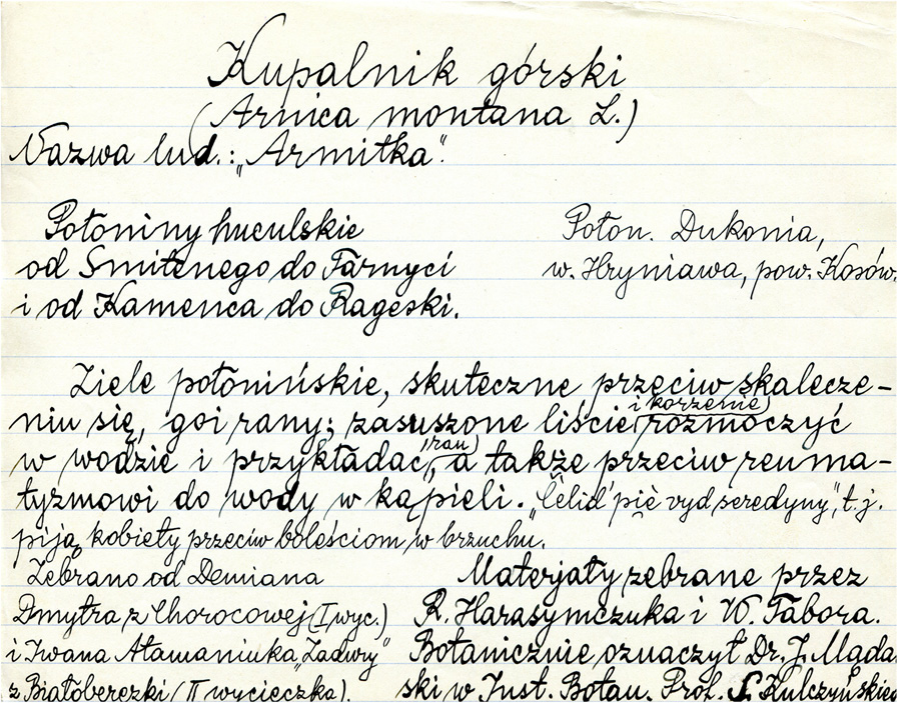
An exemplar filecard with information on plant names, uses and botanical identification, from a Hutsul village (Hryniawa)

The source of data for this article is the information gathered by professor Adam Fischer’s coworkers in the field between 1929–1934; handwritten and stored on filecards. The whole collection of the material, which had been prepared for the *Lexicon of Slavic beliefs and customs* contains over 6000 separate cards. They comprise both published and unpublished materials. For the purpose of this analysis we chose only unpublished data. The cards containing published and unpublished material may easily be distinguished, as they have different layout and content. The cards with published material comprise information about plant species names (common and sometimes local), plant use and the reference. In contrast, cards comprising unpublished material include the collection site, county and province (usually at the top of the card, beneath the Latin, common or local names or combination of them), the plant’s use and the name of the collector or alternatively the name of the *Koło Krajoznawcze* [Tourist Club]. In the cases of species identified by a botanist, additional information was provided – who determined the taxon and where (Fig. 2). The data from the unpublished filecards were entered into Microsoft Excel Spreadsheets for further analysis. We included all the possible information found on cards: all names (Latin, common, local), plant parts used, specific uses, forms of preparation in the case of medicinal, veterinary and food plants, as well as the locality, county and province the information was gathered from. The data set from the Ukraine comprises 290 filecards. Sometimes different cultural uses were lumped together on a single card and sometimes they were split.

### Botanical identification

Out of 179 plant taxa whose uses were registered for the study region, 106 were identified in the Botanical Institute of the Jan Kazimierz University (JKU) in Lviv. More than half of them include an additional annotation stating that it was Dr. Józef Mądalski from the Botanical Institute of JKU who identified the taxa. Therefore, nearly 60 % of the plant taxa described here should be reliably identified. All the Latin names provided by botanists from the JKU were verified according to the Plant List. Other Latin names were added by us, estimations based on common Polish names and local names – as some of the filecards contained both of them, and some only one of them. The estimated names were then cross-checked with the Polish and Ukrainian ethnobotanical and ethnographic literature [8, 15, 39–41]. For example, we have information that seeds of “arbuz” identified as *Citrullus* sp. were used as fodder and in oil production. This information raised our suspicion, as other sources report pumpkin seeds (*Cucurbita pepo*) to be used for this purpose. We checked, especially, Rokossowska’s data from similar territories gathered in the late 19^th^ century [8]. Rostafiński also wrote about the common mistake made by ethnographers who confused *Cucurbita* with *Citrullus* [15]. On several occasions, we were not sure of the correctness of identification, thus we put a question mark (?) next to it.

### Data analysis

The collected material was divided into use categories: medicinal, ritual, animal wellbeing (fodder and veterinary), food, ornamental, apotropaic and household. The latter is a heterogeneous group which could be also named “technology”. These categories were rather *etic* constructions, which took into account classical divisions from European ethnography [42]. These groupings enabled us to apply the Use Value index proposed by Prance et al. [43]. According to this index, the use value of species can be measured without knowing the frequency of citation by informants. Originally, Prance and his collaborators evaluated species’ use value by giving 1.0 point for major uses and 0.5 for minor uses. However, in the case of historical data, without having access to accompanying interviews and fieldnotes, which could give more insight into species use importance, it is difficult to estimate which uses are more or less important for the resilience of a given culture. Therefore, in our study each use of a plant taxon was counted as 1.0. Some species had different uses within one category – these were counted as separate uses. Then, for each taxon, we summed up the values corresponding to its uses.

The second index of cultural importance we applied was used only for medicinal plants. This was the Relative Importance value proposed by Bennett and Prance [44], which was designed to measure medicinal plant versatility. It takes into account two factors: the relative number of body systems (RelBS) treated with a given plant taxon and the relative number of pharmacological properties (RelPH) ascribed to this species. Therefore, this index was appropriate for our set of data, however in some cases we had to hypothesize the pharmacological properties of plant taxa as we usually had information on the illnesses treated with these plants, but not the pharmacological actions they produced. For example, a folk illness called “wzruszenie” was associated with muscle strain after lifting something heavy. In this context we perceived the role of *Campanula patula* subsp. *abietina,* applied in the treatment of “wzruszenie,” as analgesic, but it could be also considered a relaxing agent. In this situation we had to take an arbitrary decision.

The analysis was done twofold, both on the plant species level and the use level. On the species level we took into account taxa used for a given cultural use (e.g. wound healing, wild foods, construction, cattle fodder, and so forth), on the use level we took into account separate uses of a given taxon defined as: a plant part “p” of a species “s” used for “u” in a locality “l”.

## Results

### General findings

In total, the examined filecards contained information about 179 plant taxa still used or only remembered in different spheres of peasant culture in the 1930s in the western Ukraine. For 179 botanicals 324 different uses were recorded. The species which achieved the highest Use Value were: yarrow (*Achillea millefolium*) (7), garlic (*Allium sativum*) (6), lesser periwinkle (*Vinca minor*) (6), St. John’s wort (*Hypericum* sp.) (5) and juniper (*Juniperus communis*) (5) (Appendix).

Among the collected plant names, Polish names dominate (59 %, 96 out of 163) over clearly Ukrainian and Ruthenian ones (31 % of names). The remaining 10 % of names had unclear origins or could be used by both groups.

### Medicinal plants

Medicinally useful plants represent the largest category of uses. We registered 87 taxa and 138 separate uses of plants employed in the treatment of 13 different body systems. The most frequent were the digestive tract and respiratory systems, for which we found 24 uses of plants, respectively, then skin (23), musculoskeletal (17), “symptoms” which include headache and fever (13) and reproductive and urinary systems (9 uses each). The most frequently treated illnesses in the study region, according to the number of taxa, were: coughs (18 different taxa), then “zawianie” (the effect of draughts, e.g. stiff neck) (10), wounds (9), stomach ache (9), headache (7) and women’s gynecological problems (4).

Plant taxa which scored the highest Relative Importance value and exhibited the most versatile medicinal applications were: *Achillea millefolium* (100) and *Tussilago farfara* (100) – both used in the treatment of four body systems and recognized for four different pharmacological actions. Other versatile taxa were: *Veratrum album* (87.5), *Allium sativum* (75) *Artemisia absinthium* (75), *Viola tricolor* (75), and to a lesser extent: *Arnica montana* (62.5), *Hypericum* sp. (62.5), *Juniperus communis* (62.5) and *Matricaria chamomilla* (62.5).

The filecards sometimes lacked information about the plant parts used, or the modes of preparation of medicinal plants. Nonetheless, we can hypothesize that leaves (38 uses) and stalks (24 uses) were most often employed in home phytotherapy in Eastern Europe. Other plant parts were used rather sparsely: flowers and roots (8 uses each), bulbs (6), fruits (5) and seeds (3).

Although we do not have information about the forms of preparation and application for 47 plant uses, amongst the known forms of application external uses (51) predominated over internal ones (41). The external uses included baths, compresses and fumigations, sometimes combined with charm healing. They were not confined to skin problems, muscular pains and toothache – normally healed in this way, but also used to treat fever, headache, kidney problems, nervous tension and children’s rachitis (also called “English illness”). A prevalence of herbal remedies comprising only one ingredient was also observed in the study region. Occasional mixtures used externally were characterized by a low number of herbal and secondary components, such as honey, vegetable oil, eggs, and alcohol. In this fashion, *Chelidonium majus* was mixed with olive oil, fir resin and beeswax to treat pustules; *Cichorium intybus* was mixed with *Galium verum* in a compress to treat kidney problems; *Plantago major* was macerated in alcohol for bites and stings; *Thymus pulegioides* was applied in tincture with iodine to heal “rotting legs”; cf *Tussilago farfara* was prepared in ointment with eggs and spirit to treat aches and pains. We managed to find information on three different mixtures used internally: *Cetraria islandica* was soaked in milk to treat coughs; *Persicaria bistorta* was macerated in vodka and used for vaginal bleeding by Hutsul women; and *Thymus* sp. was prepared in an infusion with *Tanacetum vulgare* for blood cleansing. Characteristically, no pharmaceuticals were combined with plant medicines, or at least the field workers did not report this practice.

### Plants used in rituals and ceremonies

The data in this category comprises at least 85 species of plants. The most frequently mentioned plant is *Vinca minor,* used for both wedding and funeral ceremonies, and for blessing Assumption Day bouquets or Corpus Christi wreaths. Most data in this use category are plants blessed in churches on Assumption Day. This tradition is present in both Roman-Catholic (Polish) and Greek-Catholic (Ukrainian) churches, hence the presented data may concern both denominations. Many of the plants blessed on Assumption Day in the present territory of Ukraine are the same as those blessed in the Polish part of the Carpathians (e.g. *Papaver somniferum, Achillea millefolium, Centaurea jacea, Mentha* spp., *Eupatorium cannabinum,* apples, carrot roots; for the list of studies on Assumption Day bouquets see [45–47]. *Aconitum* x *cammarium* is mentioned in as many as three places. The genus *Aconitum* is rarely blessed in Poland (almost only in the Tatra Mts; Ł.Ł. personal observations).

### Animal wellbeing

This category embraces both plants that were used for fodder (10 species, 11 uses) and veterinary purposes (8 species, 9 uses). In the fodder category, two species stand out, which were added to cattle fodder in order to “produce good quality cream”, namely *Agrimonia eupatoria* locally known as “smetannyk” [*smetana* means cream in Ukrainian] and *Aquilegia vulgaris*. Another interesting use, called “woroniacze masło” [literally *crow’s butter*] as ascribed to cf *Sedum telephium* L. Its roots were cooked with salt and given to cows so they produced more milk. Another species used for enhancing milk production was *Gentiana asclepiadea*, but only herbs from blessed wreaths were considered for this purpose. Other plants were added to fresh green fodder, e.g. *Brassica rapa*, and nettle (*Urtica* spp.) or simply to cattle fodder: beetroot (*Beta vulgaris*), pumpkin (*Cucurbita* sp.), garden pea (*Pisum sativum*) and oak (*Quercus*) fruits were mentioned as typical pig fodder.

In the study region, peasants applied different botanicals to prevent cow, horse, pig and poultry illnesses, as well as to treat them. Cows which had just given birth were fumigated with the blessed herb of *Vinca minor* and the same blessed species was added to their fodder. Different plants were also used to treat internal infections (*Tanacetum vulgare*, *Valeriana officinalis*), swollen intestines (*Amaranthus caudatus*), and for parasites (cf *Veratrum* sp.). For liquid retention, parsley (*Petroselinum crispum*) was used, and also applied for humans due to its recognized diuretic properties. Horseradish (*Armoracia rusticana*) was the base of the treatment of one particular illness which affected horses, namely scrofula. It was grated and mixed with oats. In some cases, it was difficult to decide if a given plant species was more a medicine or fodder, as most of them were given in the form of fodder, especially those applied internally, and direct therapeutic use was reported only for a few species.

### Other uses

Fifteen cultivated and wild species were mentioned as food plants. In this category, two species were cited as ceremonial food: *Carthamus tinctorius,* added to wedding cakes, and wheat (*Triticum aestivum*) – an ingredient for a Christmas dish called “kutia” and for a St Andrew’s and Easter dish called “paski”. Three taxa were used for making oil in village oil mills: hemp (*Cannabis sativa*), pumpkin seeds (*Cucurbita* sp.), and flax (*Linum usitatissimum*). Gruel was prepared from pumpkin seeds, barley (*Hordeum vulgare*), and plantain (*Plantago lanceolata*), the latter mixed with clover seeds for this purpose. Just three species were mentioned as eaten for their fruits: hazel (*Corylus avellana*), plum (*Prunus domestica*) – cultivated near houses, and *Ribes uva-scrispa* – appreciated by children as a snack. Birch sap (*Betula* sp). was left for 3–4 months to produce vinegar. Neither famine plants nor species used as emergency food in periods of shortage were mentioned. Overall, the food plants represent an eclectic collection of very diverse uses, and together with the small number of taxa mentioned, give an impression of field data which were not systematically collected within this use category.

Generally, we can learn very little about plant management and plant perception from the information stored on the filecards. For this reason, we noted all the plant species that were mentioned as cultivated in homegardens. Eight different species were reported as grown in peasant homegardens. Most of them performed diverse cultural functions, however in a few cases it is difficult to establish their use beyond their ornamental value (e.g. *Aster* sp., *Hyssopus officinalis*). *Aconitum* × *cammarum* was used as an adornment by young girls and kept in gardens to ward off the Devil. It was also blessed during Corpus Christi and on Assumption Day. Other species cultivated in homegardens that were blessed in church ceremonies were marigold (*Calendula officinalis*), opium poppy (*Papaver somniferum*) and rue (*Ruta graveolens*). One species – *Dracocephalum foetidum* – was considered as a natural insect repellent and burned in pots and pipes.

The filecards contain very little information that could be considered ethno-ecological observations, e.g. abbreviated forms or parts of some legends and stories, which may have fulfilled educational and mnemonic purposes in the process of ecological knowledge transmission. For example, the following observation was transcribed about *Valeriana officinalis,* called “wodolon ogrodowy”: “Wodolon grows in the company of toja [*Aconitum*], and if toja is not around, wodolon cannot grow for long. They are an enchanted pair of lovers, cursed by their parents, who did not let them marry.” We also learn why elder (*Sambucus nigra*), considered medicinal plant, was also perceived as the Devil’s plant and approached carefully. “Its berries used to be edible, but since Judas hanged himself it is considered as cursed by God and is harmful. Its cutting may cause illness, death, or insanity”.

Although some species were considered to be loaded with sinister powers, like the aforementioned elder, others were kept close to households or to the body as a form of protection against these evil forces. One of these was the aforementioned *Aconitum* × *cammarum*, others were *Angelica sylvestris*, nettle and garlic.

Finally, very few species were cited as used for household or technological purposes, providing very fragmentary knowledge on this part of material culture. At the time of the fieldwork, clothes were still made from hemp (*Cannabis sativa*) and flax (*Linum usitatissimum*). The only mentioned dyeing plant was *Carthamus tinctorius,* used for yellow colouring. For construction purposes, only hardwood tree species were cited, i.e. beech (*Fagus sylvatica*) and oak (*Quercus* sp.) As material for weaving baskets, reed (*Phragmites australis*) was reported, and *Euonymus* sp. was used by cobblers to make pegs.

## Discussion

### How could the pre-selection of topics affect the data set?

It is a well known ethnographic fact that the kind of questions we ask in the field influence the answers we obtain. Hence, the first issue is whether there is a correlation between the questions’ scope (proposed by Fischer) and the diversity of answers obtained within this research. We shall take into account the first questionnaire published in *Lud* [34] and repeated in *Orli Lot* [35]. We may assume that the order of questions reflected their importance to Fischer. The first question was about folk names and their possible etymologies. We miss 25 local names of 179 plant species reported for western Ukraine (for those plants only uses and official names were given). Only in the case of three plant species was no information about their use obtained – just their names. Hence we can state that the field researchers were more concerned about collecting plant uses than local plant names and their etymologies. The second question was about specific uses, mentioned in the following order: food, construction, clothes, dyeing agents, medicines and poisons. The answers, however, do not match the question order. Medicinal plants outnumbered the remaining uses, and actually we could find very little information about plants used for food, construction, clothes making and dyeing. Other uses such as veterinary and fodder, although not elicited in the questionnaire, brought more responses than the above-mentioned domains. The third question, which was focused on plants with supernatural powers, produced virtually no results. The fourth question, focused on plants used in ceremonies and rituals, indeed resulted in a long and diverse list of species blessed on Assumption day and a shorter one concerning other ritual purposes. The last three issues raised by Fischer: plants as decorative motifs, as toys, and as plants in stories and songs, produced very fragmentary and eclectic data. Therefore, we may conclude that the scope of the questionnaire did not have much effect on the character of the data set obtained within the fieldwork. In other words, what had been expected, was not found. Instead, the array of species and the corresponding uses registered in the study region most probably reflected the reality: the vital role of phytotherapy in folk medicine, the importance of animal wellbeing and the crucial role of plants in church ceremonies. However, the relative lack of food plants (including staples) may be due to the concentration on wild species and the “obviousness” of the topic, rather than the small variety of food plants employed in the peasant diet.

### Whose ethnobotany and how reliable is the data?

More than half of the recorded plant names are Polish, and a third are typically Ukrainian. This prevalence of Polish plant names may stem from a few facts: most data come from the areas around Lviv, and areas west or south of it, with the highest density of Poles. As the questionnaire was in Polish, it was the Polish speaking and writing people who were more prone to answer. Moreover, some of the plant names are similar in Polish and Ukrainian, so they may have been Polonized in the writing, e.g Ukrainian “kalyna”*,* may have been changed to Polish “kalina”*.* If this is indeed mainly an ethnobotany of the Polish population in Ukraine, the data cannot be compared with the present state, as most of the descendants of the people whom it concerns are now dispersed in western and northern Poland.

Searching through the materials we found several doubtful identifications, particularly associated with the filecards identified by Prof. Mądalski. These were cases in which a given folk name was widely associated with a different taxon than the one identified. It is not unlikely that there may have been mistakes when picking specimens for identification, as it is unlikely that a professional botanist would have made such gross mistakes. We tried to eradicate doubtful data by looking at the known Ukrainian and Polish folk plant names. In the case of doubt, it was marked in the Appendix.

### For what purposes did Polish, Ruthenian and Ukrainian peasants use plants?

The general advantage of extensive ethnobotanical research, embracing the whole array of cultural uses, is that it shows in which cultural spheres plants seem important and salient. Complex findings are valuable, provided there is methodological rigor in the field data gathering (the same stress put on all the study domains, or alternatively good quality free listing). Unfortunately, in the case of historical data, without insight into the fieldwork procedures we cannot have this certainty. Therefore, the following analysis and observations do not have ultimate explanatory strength, and are, rather, our hypotheses and assumptions.

The medicinal use of plants happened to be the most salient domain in the peasant culture of western Ukraine. The character of reported health problems and their treatment suggests a symptomatic approach to illness perception and the healing process. Medicinal plants seemed to be related to naturalistic etiologies – tangible health problems, which were solved with plant applications [48]. The data also contain “regional singularities” expressed in the importance of plant remedies to treat “zawianie” (the effect of draughts) and treated with 10 different taxa. Our findings clearly show that plant species were sparsely used to treat folk illnesses [49], which may confirm the theory that in eastern European traditional medicine folk illnesses have been normally treated with charm healing and faith healing, rather than with plants [7, 11, 39, 50]. Alternatively, the broad scope of the research might have been an obstacle to an in-depth approach to this matter, and studying traditional medicine normally requires return field research and the gaining of interlocutors’ confidence. Moreover, ethnographers who were eager to register formulas of charm healing did not pay much attention to specific plant species that may have accompanied charm and faith healing [51], which might have created a bias.

Nonetheless, we can learn quite a lot about the character of plant remedies and forms of their administration. The importance of external uses in traditional folk culture is usually related to a high frequency of skin illnesses and a lack of hygiene [39]. Moszyński even wrote that “Slavs had never been fond of bathing and had not shown a passion for cleanliness in general” [42, 52]. Herbal baths, according to this author, were common for babies and toddlers, and always implied some healing action, and as children grew up baths became rarer and rarer. Our data also confirm the importance of baths among children for such illnesses as: rachitis convultions, weakness, and wound healing. However, we need more systematic diachronic studies on this matter, to be able to generate a conclusion about external medicinal use prevalence in traditional folk medicine and its slow replacement by internal uses in the 19^th^ and 20^th^ centuries. Another issue worthy of further studies is that of herbal mixtures in central and eastern Europe. According to Paluch’s review of folk phytotherapy in Poland, the use of single species predominated [40]. Occasional mixtures were made from 2–5 components (both herbal and non-herbal) and were used predominantly in the treatment of respiratory system illnesses. Our findings remain in line with these observations, with one exception – only one mixture was recorded for respiratory illnesses.

Petkevičius and colleagues, in a recent contribution analyzing archival data on medicinal plants sold in Vilnius markets, stated that medicinal use of plants persisted in the interwar period due to the relative poverty of local people who were not able to purchase synthetic drugs, and also due to the slow development of a national, free-of-charge health system [51]. Similar statements may also apply to western Ukraine. In-depth ethnomedical studies conducted by physicians in western Ukraine in the 19^th^ and the beginning of the 20^th^ centuries show an extensive home sector within folk medicine, in which phytotherapy was particularly important. Among non-biomedical specialists the most important role was played by ‘*baby’* – women healers who were also midwives, followed by bonesetters, and local healers who used both charm healing and phytotherapy. At the end of the chain of medical providers was the doctor, who peasants consulted rarely, normally when their lives were threatened [11, 39]. Our data also confirm that home phytotherapy was still a very important form of illness treatment in the study region, and perhaps plant medicines had a central role in folk medicine.

## Conclusions

The general advantage of extensive ethnobotanical research, embracing a whole array of cultural uses, is that it shows in which cultural spheres plants seem important. In contrast to the list of issues explicitly indicated in the first questionnaire by Fischer, the most salient cultural domains in the study region turned out to be medicinal, ceremonial and related to animal wellbeing. Further analysis, from other regions, will enable us to say whether there were regional differences in these domains or whether there was uniformity in plant use. Any potential differences should be contextualized and explained. We believe that historical data of this kind make an important contribution to diachronic ethnobotanical and ethnoecological studies and, in a wider sense, help us to elucidate how folk culture evolves.

## Appendix

List of plant species used in folk culture of eastern Galicja (Galizien), now western Ukraine, in the 1930s.

Legend: Use categories: A – apotropaic, B – beliefs, C – cultivated, F – food, Fd – fodder, M – medicinal, NS – not specified, R – ritual, T – technology, V – veterinary. Latin names in square brackets are the original names found on filecards.

*Achillea millefolium* L. Asteraceae – **M**: bleeding wounds, lubrication with juice (Czyszki, Bratkowice, Ostróg); dysentery (Czyszki); menstrual pain, nose bleeding, postpartum (Ostróg); pustules, ointment with flour (Sokolniki); **R**: blessed on Assumption Day (Kniażdwór, Sopów); LN: krwawnik (Czyszki, Kniażdwór, Ostróg, Sopów), derewyj białogłowy (Sokolniki), derewec (Bratkowice)

*Aconitum* × *cammarum* L. Ranunculaceae – **A & C**: cultivated in homegardens, protects against the Devil (Babin, around Łuck); **O**: girls put in their hair; **R**: blessed on Corpus Christi (around Łuck), blessed on Assumption Day (Koniuchy, Tarnopol); LN: toja (Babin, around Łuck), tojad (Koniuchy, Tarnopol)

*Agrimonia eupatoria* L. Rosaceae – **Fd**: added to cows’ food in order to produce good quality cream (Stary Sambor); **R**: blessed on Assumption Day (Stary Sambor); LN: smetannyk

*Agrostis canina* L. Poaceae – **NS**: it grows in the area (Sławsko); LN: stebłycia

*Agrostis capillaris* L. Poaceae – **R**: blessed on Assumption Day (Sopów); LN: nd

*Alectorolophus* sp. Orobanchaceae – M: stomach-ache, soaked in water (Hryniawa); LN: świczkie, dzwonoczok

*Allium cepa* L. Amaryllidaceae – **M**: wounds, compress; cough, syrup with sugar (Dublany); LN: cebula

*Allium sativum* L. Amaryllidaceae – **A**: blessed garlic used to rub crosses (Stary Sambor); “When a young girl is traveling to another village she should carry garlic with herself, it will protect her against evil forces” (around zadniestrzańskie); **M**: cholera, cough, dysentery, typhoid fever (in all cases with blessed garlic) (Stary Sambor); LN: czosnek

*Allium sybiricum* L. Amaryllidaceae – **NS**: it grows in the area (Hryniawa); LN: dyka cebula

*Alnus* sp. Betulaceae – M: wounds (Dublany); LN: olcha

*Amaranthus caudatus* L. Amaranthaceae – **M**: abdominal pain; **R**: blessed on Assumption Day; **V**: swollen intestines (Stary Sambor); LN: dzikie proso

*Amaranthus hybridus* L. Amaranthaceae – **R**: blessed on Assumption Day (Koniuchy); LN: dyke proso

*Angelica* sp. Apiaceae – **R**: blessed on Assumption Day (Stary Sambor); LN: dzingol

*Angelica sylvestris* L. Apiaceae – **A**: protects against illnesses when blessed and caried closed to the body (Stary Sambor); LN: dzingol

*Anthoxanthum odoratum* L. Poaceae – **R**: blessed on Assumption Day (Kniażdwór, Sopów); LN: nd

*Anthyllis vulneraria* L. [*Anthyllis kerneri* Sagorski] Fabaceae - **R**: blessed on Assumption Day (Kniażdwór, Sopów); LN: nd

*Aquilegia* sp. Ranunculaceae - **R**: blessed on Assumption Day (Koniuchy); LN: toja

*Aquilegia vulgaris* L. – **Fd**: added to cows’ food in order to produce good quality cream (Babin); LN: rostópad [this name is usually applied to *Chelidonium majus* L., so this data should be treated with reserve]

*Arctium* sp. [*Petasites* sp.] – **M**: fever, compress (Bratkowce); LN: łopuch

*Armoracia rusticana* P.Gaertn., B.Mey. & Scherb Brassicaceae – **M**: headache; **V**: scrofula (horse illness), grated and mixed with oats (Dublany); LN: chrzan

*Arnica montana* L. Asteraceae – **M**: rheumatism, baths; women’s abdominal pain; wounds, compress (Hryniawa); LN: armitka

*Artemisia abrotanum* L. Asteraceae – **R**: blessed on Assumption Day (around Łuck); LN: boże drzewko

*Artemisia absinthium* L. Asteraceae – **M**: ague, infusion (Loposzym); stomach cramps, macerated in water (Dublany); “zawianie” (effect of draught, e.g. stiff neck), infusion or decoction (Chyrów); LN: piołun

*Artemisia annua* L. Asteraceae – **R**: blessed on Assumption Day (Koniuchy); LN: polubownyk

*Asparagus officinalis* L. Asparagaceae – **R**: blessed on Assumption Day (Hołosko Wielkie, Tarnopol); LN: nd

*Aspidium* sp. Dryopteridaceae – **A**: it is placed in the four corners of a newly built hut (Dublany); LN: paproć

*Aster* sp. Asteraceae – **C**: cultivated in homegardens (Dublany, around Łuck); LN: astra, iastra (Dublany), aster, taziłki, talizki (around Łuck)

*Avena sativa* L. Poaceae – **M**: cough, infusion (Dublany); LN: owies

*Bellis perennis* L – Asteraceae - **M**: cough, infusion (Dublany); LN: stokrotka

*Beta vulgaris* L. Amaranthaceae – **F**: soup, called “barszcz”; **Fd**: cattle fodder (Dublany); LN: burak

*Betula* sp. Betulaceae – **F**: vinegar; **M**: cough, warmed up sap (Dublany); LN: brzoza

*Borago officinalis* L. Boraginaceae – **R**: blessed on Assumption Day (Hołosko Wielkie); LN: nd

*Brassica oleracea* L. Brassicaceae – **M**: wounds, compress (Czyszki); LN: kapusta

*Brassica rapa* L. Brassicaceae – **Fd**: added to fresh green fodder (Dublany); LN: rzepa

*Bupleurum rotundifolium* L. Apiaceae – **R**: Aspergillum is made for priests, who use it to scatter over the dead (Jasionów Górny); LN: taśkavec

*Calendula officinalis* L. Asteraceae – **C**: cultivated in homegardens (around Łuck); **R**: blessed on Assumption Day (Hołosko Wielkie); LN: nagietek

*Campanula glomerata* L. Campanulaceae – **M**: stomach ache, soaked in water (Hryniawa); LN: dzwonoczok, świczkie

*Campanula patula* subsp. *abietina* (Griseb. & Schenk) Simonok. Campanulaceae – **M**: “wzruszenie” (a folk illness) (Hryniawa); LN: pydójma

*Cannabis sativa* L. Cannabaceae – **F**: oil; **T**: cloths’ making (Dublany); **R**: blessed on Assumption Day (Sopów); LN: konopie

*Carduus acanthoides* L. Asteraceae – M: against ‘podużanie’ [a folk illness]. Dried herb is thrown into a fire, together with blessed incense, and it is said: “If it turned badly, change it to good”, then the sign of the cross is made three times (Bratokowice); LN: bodiak kłujący

*Carlina acaulis* L. or *Inula helenium* L. ? Asteraceae – **M**: nervous tension, baths (Sławsko); LN: dewiatosył

*Carthamus tinctorius* L. Asteraceae - **F**: additive to a wedding cake; **T**: dyes yellow (Podole); **R**: blessed on Assumption Day (Tarnopol); LN: nd

*Carum carvi* L. Apiaceae – **R**: blessed on Assumption Day (Sopów); LN: nd

*Centaurea jacea* L. Asteraceae – **R**: blessed on Assumption Day (Kniażdwór); LN: nd

*Centaurium erythraea* Rafn Gentianaceae – **M**: stomach ache, infusion (Olchowiec (?), Łopuszna); LN: centuria

*Cetraria islandica* L. Parmeliaceae – **M**: cough, soaked in milk (Hryniawa); LN: hrań

*Chelidonium majus* L. Papaveraceae – **M**: foot callus, compress; jaundice, infusion (Bratkowice); pustules, ointment with olive oil, fir resin, beeswax (Sokolniki); LN: dziczyzna (Bratkowice), jaskosz (Sokolniki)

*Cichorium intybus* L. Asteraceae – **M**: kidney problems, compress with *arnik* (*Galium verum L*.) (Sokolniki); toothache, smoked in a pipe (Stary Sambor); **R**: blessed on Assumption Day (Stary Sambor); LN: dorożnyk (Stary Sambor), dzwonik (Sokolniki).

*Cucurbita* sp. [*Citrullus* sp.] Cucurbitaceae – **F**: seeds boiled with milk, called kasha; oil; **Fd**: cattle fodder (Dublany); LN: arbuz

*Convolvulus arvensis* L. Convolvulaceae – **R**: blessed on Assumption Day (Sopów); LN: nd

*Coriandrum sativum* L. Apiaceae – **C**: cultivated in homegardens (Babin); **M**: headache, compress (Babin); “zawianie” (effect of draught, e.g. stiff neck), fumigation (Sozań); LN: koléndra

*Corylus avellana* L. Betulaceae – **F**: food (not specified) (Dublany); **M**: wounds in children, baths with blessed branches (Stary Sambor); **R**: blessed on Assumption Day (Stary Sambor); LN: laszczyna, laskowe orzechy

*Crataegus* sp. Rosaceae – **M**: stomach ache, infusion (Dublany); LN: głóg

*Crepis* sp. Asteraceae – **R**: blessed on Assumption Day (Kniażdwór); LN: pępawa

*Cyanus segetum* Hill Asteraceae – **B**: used by water-nymph as adornment (Wołyń); LN: bławat

*Cynosurus cristatus* L. Poaceae - **R**: blessed on Assumption Day (Kniażdwór); LN: grzebienica pospolita

*Cytisus* sp. Fabaceae – **M**: jaundice, infusion (Sokolniki); LN: wierzba jedwabna

*Dahlia* sp. Asteraceae – **R**: blessed on Assumption Day (Koniuchy, Tarnopol); LN: georginja

*Daucus carota* L. Apiaceae – **R**: blessed on Assumption Day (Tarnopol); LN: nd

*Dianthus caryophyllus* L. Caryophyllaceae – **R**: blessed on Assumption Day (Hołosko Wielkie); LN: goździk

*Dianthus sylvestris* Wulfen ? Caryophyllaceae – **R**: blessed on Assumption Day (Sopów); LN: goździk leśny

*Dipsacus* sp. Caprifoliaceae – **M**: “zawianie” (effect of draught, e.g. stiff neck), fumigation (Sozań); LN: szczeć

*Dracocephalum foetidum* Bunge Lamiaceae – **C**: cultivated in homegardens; **M**: against fright (folk illness), fumigation; **T**: natural repellent burned in pots and pipes (Jasionów Górny, Sokołówka); LN: samnosy, samaśin

*Drosera* sp. Droseraceae – **R**: blessed on Corpus Christi (around Łuck); LN: rosiczka

*Dysphania botrys (L.) Mosyakin & Clemants* [*Chenopdium botrys* L.] Amaranthaceae – **M**: headache, bath; illness prevention, bath (Jasionów Górny); LN: mȳrzile

*Elymus repens* (L.) Gould Poaceae – **R**: blessed on Assumption Day (Kniażdwór); LN: perz

*Epilobium angustifolium* L. Onagraceae – **M**: “zawianie” (effect of draught, e.g. stiff neck), fumigation (Sozań); LN: nd

*Equisetum arvense* L. Equisetaceae – **M**: common cold, infusion (Sokolniki); LN: sosenka polna

*Equisetum fluviatile* L. Equisetaceae - **M**: urinary retention, infusion (Sokolniki); LN: sosenka moczarowa

*Equisetum palustre* L. Equisetaceae – **R**: blessed on Assumption Day (Sopów); LN: skrzyp

*Equisetum variegatum* Schleich. ex F. Weber & D. Mohr Equisetaceae – **R**: blessed on Assumption Day (Kniażdwór); LN: skrzyp

*Euonymus* sp. Celastraceae – **T**: cobblers make pegs (Wołyń); LN: proskurzyna

*Eupatorium cannabinum* L. Asteraceae – **M**: women’s gynecological problems; **R**: Blessed on Assumption Day (Stary Sambor); LN: prystrit

*Fagopyrum esculentum* Moench Polygonaceae – **M**: stomach ache, poultice (Sołonka Mała); LN: hreczka dzika

*Fagus sylvatica* L. Fagaceae – **T**: house furniture; wagon and mill wheels; stove firewood (Dublany); LN: buk

*Festuca* sp. Poaceae – **R**: blessed on Assumption Day (Kniażdwór); LN: kostrzewa

*Fraxinus excelsior* L. Oleaceae – **R**: decoration of houses for Whit (Dublany); LN: jesion

*Galium verum* L. Rubiaceae – **M**: kidney problems, compress with dzwonik (*Cichorium intybus* L.) (Sokolniki); **R**: blessed on Assumption Day (Kniażdwór, Sopów); LN: arnik (Sokolniki)

*Genista tinctoria* L. Fabaceae – **R**: blessed on Assumption Day (Koniuchy); LN: janowiec

*Gentiana asclepiadea* L. Gentianaceae – **Fd**: cows’ food from blessed wreath, in order to produce more milk; **R**: blessed on Corpus Christi (Stary Sambor); LN: świcznik

*Geum montanum* L. Rosaceae – **M**: “wzruszenie” (a folk illness) (Hryniawa); LN: pydójma

*Helianthus annuus* L. Asteraceae – **R**: blessed on Corpus Christi (around Łuck); blessed on Assumption Day (Tarnopol); LN: słonecznik

*Helianthus tuberosus* L. Asteraceae - **R**: blessed on Corpus Christi (Stary Sambor); LN: topinambór

*Rumex* sp. [*Heracleum sphondylium* L.] Polygonaceae – **F**: eaten raw, ocasionally in a soup, called “barszcz” (Dublany); LN: kwasek

*Hordeum vulgare* L. Poaceae – F: gruel (Dublany); LN: jęczmień

*Hypericum perforatum* L. Hypericaceae – **R:** blessed on Assumption Day (Sopów); LN: nd

*Hypericum* sp. Hypericaceae – **M**: blood cleansing, infusion; liver problem, infusion (Sokolniki); LN: świętojańskie ziele, świętego Jana krew

*Hypericum* sp. [*Hypericum perforatum* L.] Hypericaceae – **M**: dysentery, decoction with the blessed herb; **R**: blessed on Assumption Day; **V**: bleeding cattle (Stary Sambor); LN: krowawnyk

*Hyssopus officinalis* L. Lamiaceae – **C**: cultivated in homegardens (Podole); LN: józefek

*Inula britannica* L. Asteraceae – **R**: blessed on Assumption Day (Sopów); LN: nd

*Juniperus communis* L. Cupressaceae – **M**: constipation; stomach cramps; swelling; urinary retention; **R**: incense for Christmas (Dublany); LN: jałowiec

*Knautia arvensis* (L.) Coult. Caprifoliaceae – **R**: blessed on Assumption Day (Sopów), LN: nd

*Lathyrus sylvestris* L. Fabaceae – **R:** blessed on Assumption Day (Sopów, Tarnopol); LN: nd

*Linum catharticum* L. Linaceae – **R:** blessed on Assumption Day (Sopów); LN: nd

*Linum usitatissimum* L. Linaceae – **F**: oil; **T**: cloths’ making (Dublany); **M**: “zawianie” (effect of draught, e.g. stiff neck) (Sozań); LN: len

*Lithospermum officinale* L. Boraginaceae – **M**: panacea; **R**: blessed on Assumption Day (Stary Sambor); LN: tyrbycz

*Lunaria rediviva* L. Brassicaceae – **NS**: it grows in the area (Sławsko); LN: postal

*Lycopodium clavatum* L. Lycopodiaceae – **M**: lice, washing (Babin); LN: nytóta

*Malus domestica* Borkh. Rosaceae – **R**: blessed on Assumption Day (Tarnopol); LN: jabłko

*Malva alcea* L. Malvaceae – **M**: cough, infusion (Soposzyn); LN: bluz

*Malva sylvestris* L. Malvaceae – **M**: cough, decoction; **R**: blessed on Assumption Day (Stary Sambor); LN: słyz

*Malva verticillata* L. Malvaceae – **M**: cough, infusion (Czyszki); LN: ślaz

*Matricaria chamomilla* L. Asteraceae – **M**: dysentery, infusion; epilepsy, infusion (Hryniawa); **R**: blessed on Assumption Day (Tarnopol, Koniuchy); LN: rumiènok (Hryniawa), maruna (Koniuchy)

*Medicago sativa* L. Fabaceae – **R**: blessed on Assumption Day (Kniażdwór); LN: nd

*Melampyrum arvense* L. Orobanchaceae – **M**: cough (Loposzym); LN: kocie łapki

*Mentha aquatica* L. Lamiaceae – **M**: bites; swelling (Gołonka Mała); LN: mięta wodna

*Mentha* x*piperita* L. Lamiaceae – **M**: chest pains (Stary Sambor); stomach ache (Sokolniki, Stary Sambor); **R**: blessed on Assumption Day (Stary Sambor); LN: szanta (Stary Sambor)

*Mentha* sp. Lamiaceae – **M**: cough, stomach ache (Dublany); **R**: blessed on Assumption Day (Koniuchy); LN: mięta (Dublany), ładosznyk (Koniuchy)

*Myrtus communis* L. Myrtaceae – **R**: wedding ceremony (Dublany); LN: mirt

*Narcissus* sp. Amaryllidaceae – **M**: ague (Dublany); LN: narcyza, marcyza

*Nardus stricta* L. Poaceae – **NS**: it grows in the area (Hryniawa); LN: psieńka

*Nigella damascena* L. Ranunculaceae – **R**: blessed on Assumption Day (Koniuchy); LN: pawuczok

*Ononis spinosa* subsp. *hircina* (Jacq.) Gams Fabaceae – **M**: rabies, decoction (Ostróg); **R**: blessed on Assumption Day (Sopów); LN: skażyna

*Origanum vulgare* L. Lamiaceae – **M**: women’s gynecological problems, baths with blessed herb (Stary Sambor); **R**: blessed on Assumption Day (Koniuchy, Stary Sambor); LN: materycznyk (Stary Sambor), wasylok (Koniuchy)

*Paeonia officinalis* L. Paeoniaceae – **M**: children’s convulsions (Wołyń); LN: piwonja

*Panicum miliaceum* L. Poaceae – **F**: “agły” [cracked grain] (Dublany); LN: proso

*Papaver rhoeas* L. Papaveraceae – **R**: blessed on Assumption Day (Hołosko Wielkie); LN: nd

*Papaver somniferum* L. Papaveraceae – **C**: cultivated in homegardens (around Łuck); **R**: blessed on Assumption Day (Hołosko Wielkie); LN: mak

*Papaver* sp. Papaveraceae – **R**: blessed on Assumption Day (around Łuck); LN: mak

*Parnassia palustris* L. Celastraceae – **M**: women’s gynecological problems: vaginal discharge (Hryniawa); LN: nd

*Persicaria bistorta* (L.) Samp. Polygonaceae – **M**: strong menstrual bleeding, dried and soaked in vodka (Hryniawa); LN: krywe zile

*Petroselinum crispum* (Mill.) Fuss Apiaceae – **M**: diuretic, decoction; **F**: refreshing drink, vegetable; **V**: diuretic, decoction (Dublany); LN: pietruszka

*Phalaris arundinacea* L. ‘Zebrina’ Poaceae – **R**: blessed on Corpus Christi (around Łuck); LN: trawa turecka

*Phleum pratense* L. Poaceae – **R**: blessed on Assumption Day (Kniażdwór); LN: nd

*Phlox paniculata* L. Polemoniaceae – **R**: blessed on Assumption Day (Koniuchy); LN: zaharja, ładon

*Phlox* sp. Polemoniaceae - **R**: blessed on Assumption Day (Hołosko Wielkie, Tarnopol); LN: nd

*Phragmites australis* (Cav.) Trin. ex Steud. [*Phragmites* sp.] Poaceae – **T**: weaving baskets (Dublany); LN: trościna

*Picea abies* (L.) H.Karst. Pinaceae – **M**: “zawianie” (effect of draught, e.g. stiff neck), fumigation (Sozań); LN: świerk

*Pinus sylvestris* L. Pinaceae – **M**: cough, syrup from green cones (Sołonka Mała); LN: sosna

*Pisum sativum* L. Fabaceae – **Fd**: cattle fodder (Dublany); LN: groch

*Plantago lanceolata* L. Plantaginaceae – **F**: mixed with clover seeds to make gruel (Dublany); **M**: cough, decoction (Stary Sambor); lung illness (Czyszki); **R**: blessed on Assumption Day (Kniażdwór, Stary Sambor); LN: języczki (Czyszki), babka (Dublany, Stary Sambor)

*Plantago major* L. Plantaginaceae – **M**: bites and stings, compress made from alcohol macerate (Bratkowice); festering wounds (Dublany); LN: babka

Plantago media L. Plantaginaceae – **R**: blessed on Assumption Day (Kniażdwór); LN: nd

*Poa nemoralis* L. Poaceae – **R**: blessed on Assumption Day (Kniażdwór); LN: nd

*Poa* sp. Poaceae – **R**: blessed on Assumption Day (Kniażdwór); LN: wyklina

*Podospermum roseum* (Waldst. & Kit.) Gemeinholzer & Greuter Asteraceae – **M**: headache (Hryniawa); LN: obertyn, matocznyk

*Potentilla erecta* (L.) Raeusch. Rosaceae – **M**: jaundice, pneumonia (around Stanisławów); LN: kurze łapki

*Prunus domestica* L. Rosaceae – **C & F**: cultivated for food near house (Dublany); LN: śliwa

*Prunus spinosa* L. ?? Rosaceae – **M**: dysentery, fever (Dublany); LN: cierń

*Quercus* sp.Fagaceae – **Fd**: pig fodder (fruits); **T**: building material (Dublany); **M**: lichen (gall) (Podole); LN: dąb

*Ribes uva-scrispa* L. Grossulariaceae – **F**: children’s snack (Dublany); LN: agrest

*Ruta graveolens* L. Rutaceae – **C**: cultivated in homegardens (around Łuck, Podole); **R**: blessed on Assumption Day (Koniuchy); LN: ruta

*Salvia officinalis* L. Lamiaceae – **M**: “zawianie” (effect of draught, e.g. stiff neck), fumigation (Sozań); **R**: blessed on Assumption Day (Koniuchy); LN: szitwa

*Salvia pratensis* L. Lamiaceae - **R**: blessed on Assumption Day (Sopów); LN: nd

*Sambucus nigra* L. Adoxaceae – **B**: Considered to be the devil’s tree; **M**: cough (Dublany); LN: bez czarny

*Sedum acre* L. Crassulaceae **– M:** Used to fumigate against ‘cug’ [the effect of draughts, e.g. stiff neck], it is necessary to make the sign of cross over the plant saying: “God, help this man against the evil air, which blew so unexpectedly”, the sign of cross is made again (Bratkowce); LN: rozchodnik

*Sedum* cf *acre* L. Crassulaceae – **M**: rachitis in children (“English illness”), baths (around Łuck); **R**: blessed on Corpus Christi (around Łuck); LN: rozchodnik

*Sedum maximum* (L.) Suter Crassulaceae – **M**: wounds, compress with a leaf’s pulp (Antonówka, Bratkowice); LN: kanie ziele (Antonówka), śliz maśny (Bratkowice)

*Sedum telephium* L.?? Crassulaceae – **Fd**: additive to cows’ food in order to produce more milk (Sławsko); LN: woroniacze masło

*Silene armeria* L.? Caryophyllaceae – **R**: blessed on Assumption Day (Koniuchy); LN: gwoździczky

*Sinapis* sp. or *Raphanus raphanistrum* L. Brassicaceae – **M**: “zawianie” (effect of draught, e.g. stiff neck), fumigation (Sozań); LN: gorczyca

*Stachys germanica* L. Lamiaceae – **R**: blessed on Assumption Day (Sopów); LN: nd

*Symphytum officinale* L. [*Verbascum phlomoides* L.] Boraginaceae – **M**: rheumatism, decoction with salt (Bratkowice); LN: hawies

*Tagetes erecta* L. Asteraceae – **R**: blessed on Assumption Day (Koniuchy); LN: kupczyk

*Tagetes patula* L. Asteraceae – **R**: blessed on Assumption Day; blessed on the Palm Sunday (Koniuchy); LN: czarnobryweć

*Tanacetum coccineum* (Willd.) Grierson Asteraceae – **M**: strengthening for children, bath (around Łuck); LN: maruna, panna bez posagu

*Tanacetum parthenium* (L.) Sch.Bip. Asteraceae – **M**: miscarriage, infusion (Lutonka Mała); LN: maruna

*Tanacetum vulgare* L. Asteraceae – **A**: blessed herb is burned during the storm, against thunders (Stary Sambor); **M**: blood cleansing (Sokolniki); **R**: blessed on Assumption Day (Stary Sambor); **V**: cows’ internal infections, cooked with milk (Koniuchy); LN: polne słoneczniki (Sokolniki), nawort (Stary Sambor), wodolon polny (Koniuchy)

*Thymus pulegioides* L. Lamiaceae – M: “rotting legs”, tincture of iodine and powdered herb (Hryniawa); LN: materenka

*Thymus* sp. Lamiaceae – **M**: blood cleansing, together with nawroć (*Tanacetum vulgare* L.) in infusion (around Sambor); **R**: blessed on Corpus Christi (around Łuck); LN: macierzanka

*Tilia cordata* Mill. Malvaceae – **M**: cough, infusion (Dublany); LN: lipa

*Tilia* spp. Malvaceae – **M**: cough, infusion (Sokolniki); R: decoration of houses for Whit, making crosses (Dublany); LN: lipa

*Trifolium montanum* L. Fabaceae – **R**: blessed on Assumption Day (Kniażdwór); LN: nd

*Trifolium pannonicum* Jacq. Fabaceae – **R**: blessed on Assumption Day (Kniażdwór, Sopów); LN: nd

*Trifolium* spp. Fabaceae – **M**: heart illness, compress (Czyszki); LN: koniczyna

*Triticum aestivum* L. Poaceae – **F**: ingredient for Christmas special dish called “kutia”; ingredient for St Andrew’s and Easter dish called “paski” (Dublany); **M**: “zawianie” (effect of draught, e.g. stiff neck), fumigation (Sozań); LN: pszenica

*Triticum aestivum* L. [*Triticum vulgare* Vill. var. *erythrospermum* Kornicke, var. *ferrugineum* Alef.] Poaceae – **R**: blessed on Assumption Day (Sopów); LN: pszenica

*Tussilago farfara* L. Asteraceae – **M**: cough, infusion (Sokolniki); wounds, sores, ulcers (Ostróg); LN: podbiał (Sokolniki), pidbił (Ostróg)

*Tussilago farfara* L.? [*Antennaria dioica* (L.) Gaertn.] Asteraceae – **M**: body ache, ointment made of boiled roots, 5 eggs and a glass of spirits; cough, infusion; headache, compress (Bratkowice); LN: podbiał

*Urtica* spp. Urticaceae – **A**: protects against witchcraft (Ukraine); **Fd**: added to fresh green fodder; food for poultry, mixed with potatoes; **M**: headache, compress (Dublany); LN: pokrzywa

*Urtica urens* L. Urticaceae – **M**: swelling, compress (Sokolniki, Sołonka Mała); bites (Sołonka Mała); LN: pokrzywa żegawka

*Valeriana officinalis* L. Caprifoliaceae – **B**: wodolon grows in the company of toia (*Aconitum*) and if toja is not around, wodolon cannot grow for long. It is an enchanted pair of lovers, cursed by their parents, who did not let them marry; **V**: cows’ internal infections, cooked with milk (Koniuchy); LN: wodolon ogrodowy

*Veratrum album* L. Melanthiaceae – **M**: headache, sniffed; lice, washing; nose bleeding, sniffed; pustule (Hryniawa); LN: czemeryća

*Veratrum* sp.? Melanthiaceae – **V**: worms and parasites (Dublany); LN: czymyrzyca

*Verbascum* sp. Scrophulariaceae – **M**: rachitis, baths (Sławsko); LN: dewanna

*Viburnum opulus* L. Adoxaceae – **M**: cough, blessed in wreathes (Stary Sambor); fever, infusion (Dublany); **R**: blessed on Corpus Christi (Stary Sambor); bridal wreath adorned together with barwinek (*Vinca minor* L.) (Podole); LN: kalina

*Vicia cracca* L. Fabaceae – **R**: blessed on Assumption Day (Sopów); LN: nd

*Vinca minor* L. Apocynaceae – **M**: women’s gynecological problems, baths and infusions (Podole); **R**: bride and bridesmaids used to wear as a crown on their heads; blessed on Assumption Day; placed in coffins; wreaths made for dead maidens and children (Dublany); blessed on Corpus Christi (around Łuck); during wedding, in a bridal wreath (Podole); **V**: blessed herb added to fodder for cows which have just given birth; umigation of cows after calving with blessed herb (Dublany); LN: barwinek

*Viola* sp. Violaceae – **R**: Easter table decoration (Dublany); LN: fiołek, fijułka

*Viola tricolor* L. Violaceae – **M**: blood cleansing; pneumonia; tonic (Sambor area); LN: bratek trójkolorowy

*Zea mays* L. Poaceae – **R**: blessed on Assumption Day (Kniażdwór, Sopów); LN: kukurydza
